# Biological Properties of Recently Described Wild Bramble *Rubus oklejewiczii* against the Species from Similar Niches

**DOI:** 10.3390/foods13020337

**Published:** 2024-01-21

**Authors:** Dorota Grabek-Lejko, Mateusz Wolanin, Aleksandra Szpytma, Danuta Pajda, Michał Miłek, Czesław Puchalski

**Affiliations:** 1 Department of Bioenergetics, Food Analysis and Microbiology, Institute of Food Technology and Nutrition, University of Rzeszow, Zelwerowicza 4 Street, 35-601 Rzeszow, Poland; saleksandra592@gmail.com (A.S.); danpaj81@gmail.com (D.P.); cpuchalski@ur.edu.pl (C.P.); 2 Institute of Biology, University of Rzeszów, Zelwerowicza 4 Street, 35-601 Rzeszow, Poland; mwolanin@ur.edu.pl; 3 Department of Chemistry and Food Toxicology, Institute of Food Technology and Nutrition, University of Rzeszow, Ćwiklińskiej 1a Street, 35-601 Rzeszow, Poland; mmilek@ur.edu.pl

**Keywords:** bramble, blackberries, antibacterial, antiviral, antioxidants, fruits, leaves, crops

## Abstract

The aim of this study was to compare the biological properties, such as antiviral, antibacterial, and antioxidant, of recently described pentaploid species *Rubus oklejewiczii* with tetraploid taxa growing in similar habitats including *R. plicatus*, *R. gracilis*, and *R. wimmerianus*. The antiviral potential was analyzed against bacteriophages with different genetic material: phi6 (a surrogate for the SARS-CoV-2 virus), T7, phiX174, and MS2. Antibacterial properties of fruit and leaf extracts were determined against *Staphylococcus aureus*, *Bacillus cereus*, *Escherichia coli*, and *Salmonella enterica*. The total phenolic content, as well as anthocyanins, ascorbic acid, pH, and antioxidant properties (FRAP and DPPH) were determined. *R. oklejewiczii* leaf extract was characterized by the weakest antibacterial and antiviral properties, which was closely correlated with the lowest content of polyphenolic compounds and antioxidant properties. The strongest biological properties were observed for *R. wimmerianus* leaves. Fruit extracts were characterized by lower phenolic content and antioxidant activities than leaves, with the lowest values observed for *R. oklejewiczii* extract. The antibacterial properties of fruit extracts were strongest for *R. gracilis*. The strongest antiviral potential was observed for *R. oklejewiczii* and *R. wimmerianus* fruit extracts against the bacteriophage phi6, which correlated with the lowest pH and the highest ascorbic acid content. The positive effect of the higher ploidy of *R. oklejewiczii* for most of the analyzed biological properties was not observed except for the antiviral potential of fruit extract. Due to its large and tasty fruits, this species seems to be very promising for cultivation and attractive for consumers, even though most of its biological properties were not any better compared to other examined tetraploid species.

## 1. Introduction

In Poland, blackberries are grown on a large scale and are well known and widely consumed [1]. Fruits of brambles can be collected both from the wild growing plants as well from crops. Poland is a leading exporter of blackberry fruits [2]. Blackberry fruits are known for their good taste, pleasant aroma, and nutritional value. They are eaten fresh or processed into ingredients of many dishes—jams, ice creams, desserts, bakery products, salads, and drinks. Due to its significant health-promoting properties, blackberry fruits are classified as so-called “superfruits”. Brambles are known to exhibit a wide range of pharmacological properties, and they have long been traditionally applied for their antiseptic, antimicrobial, cardioprotective, and antioxidant properties [3,4,5].

The history of the use of blackberries in traditional medicine in Europe dates back to the sixteenth century, when they were used to treat throat and eye infections [6]. Blackberry fruits and leaves are a rich source of polyphenolic compounds that have strong antioxidant properties and can significantly affect human health, reducing the risk of cancer and cardiovascular diseases, as they have antiproliferative and anti-inflammatory properties. The fruits are also rich of anthocyanins and ellagitannins which have high antioxidant potential [3].

Blackberry leaves have many uses in traditional medicine. According to Flora Health, they are consumed in the form of tea, or used as a mouthwash and gargle solution. The leaves are officially approved in Germany for the treatment of mild inflammation of the mucous membranes of the mouth and throat, for the relief of sore throat, and for the treatment of oral caries and gingivitis [7]. Many phenolic compounds, with high antioxidant properties, were previously identified in selected wild *Rubus* species by Oszmiański et al. [7,8].

There are approximately 750 bramble species in Europe [9]. In Poland, 108 bramble species occur in the wild [10]. Species can differ in their pro-health properties due to differences between the antioxidant potential of fruits and leaves [7,8]. In 2020 a new species *Rubus oklejewiczii* Wolanin & M.Nobis (section *Rubus*) was described from south-eastern Poland [11]. It is a pentaploid plant morphologically most similar to the widespread North-West and Central Europe species *R. plicatus* Weihe & Nees.

Polyploidy is a type of mutation that increases the number of chromosomes and plays an important role in the diversification and speciation of plants. In plants possessing multiple sets of chromosomes, the sizes of flowers, leaves, fruits, and seeds are often increased [12]. Moreover, the production of substances responsible for bioactive properties can also be increased [13]. *R. oklejewiczii* is a pentaploid plant [11], so our hypothesis was that this species can express higher biological properties (like antiviral, antibacterial, and antioxidant) in comparison with lower ploidy species. Species common in South Poland that occupy similar niches as *R. oklejewiczii* (e.g., forest edges, forest roadsides, and shrubs on overgrown wastelands), including *R. plicatus*, *R. gracilis* J. Presl & C. Presl, and *R. wimmerianus* (Sprib. ex Sudre) Sprib. [11,14,15], were selected for an analysis and comparison. Compared to the species mentioned above, *R. oklejewiczii* is distinguished by exceptionally large fruits (in nature, reaching up to 22 mm in length and 18 mm in width). Mature fruits of *R. oklejewiczii* can be described as very palatable, very similar to the taste of the fruits of the well-known species *R. plicatus*. *R. oklejewiczii* grows most often at the edges of fir and alder forests, usually in relatively rich and wet soils, and its geographical range is limited to the Carpathians [11]. *R. oklejewiczii*, like *R. wimmerianus* and *R. gracilis*, produce numerous primocane stems that easily root at the apex [11], which allows the bushes to multiply quickly, however, it may lead to the overcrowding of plantings and increased crop care costs.

The aim of this study was to compare the biological properties (like antiviral, antibacterial, and antioxidant) of the recently described pentaploid species *Rubus oklejewiczii* with common, widespread tetraploid taxa growing in similar habitats, such as *R. plicatus*, *R. gracilis*, and *R. wimmerianus*, and also to verify the hypothesis that a higher ploidy level of a plant has an influence on better biological properties.

According to our knowledge, the antiviral, antibacterial, and antioxidant properties of *R. oklejewiczii* were described for the first time and as a pentaploid compared to selected tetraploid taxa. Moreover, the antiviral potential of chosen brambles against bacteriophages “model organisms” used in the determination of the antiviral potential of different substances are also described for the first time.

## 2. Materials and Methods

### 2.1. Materials

**Figure 1:**
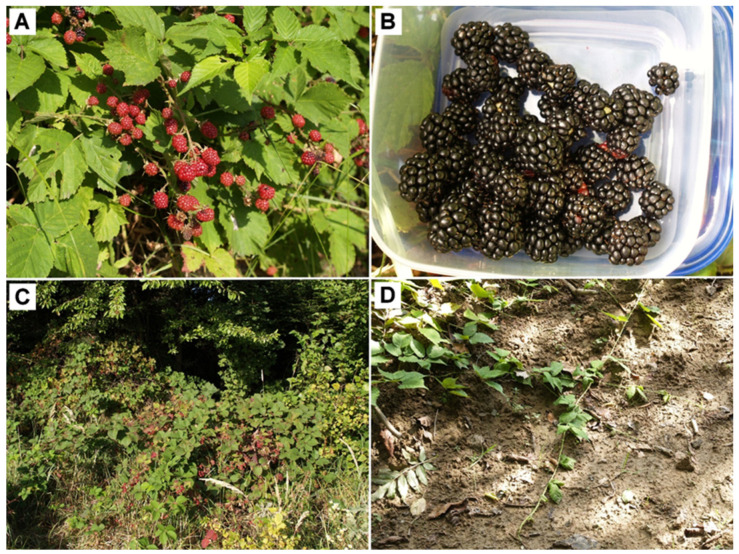
*Rubus oklejewiczii*. (**A**) infructescence; (**B**) mature fruits; (**C**) clump of bushes at the edge of fir forest; (**D**) primocane stems rooting at apex. Photos by MW.

Four different wild blackberry fruit and leaf samples were harvested at their optimum ripeness in July 2022 from closely located sites in SE Poland (Table 1). Leaf samples were taken from the middle part of primocanes (the fully developed and undamaged leaves were chosen). Species were identified by M.W. (botanist, co-author). Herbarium voucher specimens are deposited the College of Natural Sciences, University of Rzeszow, Poland. Samples of *R. oklejewiczii* were harvested in *locus classicus* (Figure 1) [11]. The leaves were dried at room temperature, in the dark for 10 days, then homogenized to a powder by using a laboratory mill (IKA 11A; BIOSAN, Vilno, Lituania). The collected powders were sieved, and the fraction with a particle size <125 µm was used to extraction. Fruits were directly lyophilized until the desired dry mass was achieved (24 h, 0.99 bar, Alpha 1–2 LD plus, Martin Christ, Osterode am Harz, Germany), then fruit powders (particle size < 250 µm) were obtained in the same manner as leaf powders. The powders were kept in a refrigerator (−70 °C) until the preparation of extracts.

### 2.2. Extraction Procedure

Three grams of powdered leaves and fruits were extracted with 30 mL of water. Extraction was carried out using an ultrasound-assisted method using an ultrasonic bath (EMAG Emmi 60 HC, Warszawa, Poland). The extraction parameters were as follows: temperature, 25 °C; ultrasound efficiency, 100% (240 W, 40 kHz); and time, 20 min. The extracts were then centrifuged (MPW-260R, Poland, 4500 rpm, time 15 min, temperature 4 °C), filtered through a paper filter (Whatman no 1), and sterilized by filtration using syringe filters (size of pores—0.45 µm). The obtained extracts were directly used for analysis or stored in the refrigerator until analysis.

### 2.3. pH Measurement

The pH value was measured in extracts using a CP 461 pH-meter (Elmetron, Warsaw, Poland).

### 2.4. Total Phenolic Content (TPC)—Folin–Ciocalteau Method

The total phenolic content was determined using the Folin–Ciocalteau method according to Grabek-Lejko et al. [4]. Briefly, 20 μL of appropriate dilutions of plant extracts were mixed with 100 μL of 10% Folin–Ciocalteau reagent and 80 μL of 7.5% sodium carbonate in microplate wells and incubated for 60 min. The absorbance was measured at 750 nm using a microplate reader (SmartReader 96, Accuris Instruments, Edison, NJ, USA). For the calibration curve, gallic acid was used, and the results were expressed as µg of gallic acid equivalents (GAE) per ml of plant extract.

### 2.5. Total Anthocyanin Content

The total anthocyanin content in fruit extracts was measured using the pH differential method as described by Grabek-Lejko et al. [4] using 10- and 20-fold diluted extracts.

### 2.6. Antioxidant Potential

#### 2.6.1. FRAP Method

Antioxidant potential was determined by the ferric reducing antioxidant power according to Grabek-Lejko et al. [4]. A FRAP solution was prepared freshly by mixing (10:1:1 *v*/*v*/*v*) 0.3 M acetate buffer (pH 3.6) and 0.01 M TPTZ (2,4,6-tripyridyltriazine) in 0.04 M HCl and 0.02 M FeCl_3_ × 6H_2_O and kept in the dark. Plant extracts, with a volume of 20 μL, were mixed with 180 μL of FRAP reagent in a 96-well microplate and incubated for 10 min away from light at 37 °C. Then, absorbance was measured at 595 nm using a microplate reader (SmartReader 96, Accuris Instruments, Edison, NJ, USA). Trolox was used for the calibration curve preparation, and the results were expressed as µmol of Trolox equivalents (TE) per ml of plant extract.

#### 2.6.2. DPPH Method

The Free-radical-scavenging ability of the extracts was tested using the DPPH radical scavenging assay as described by Grabek-Lejko et al. [4]. A test solution of the plant extract with a volume of 20 µL, was mixed with 180 µL of a 0.1 mM methanolic solution of DPPH (2,2-diphenyl-1picrylhydrazyl) and incubated at room temperature for 30 min in the dark. Then, the absorbance of the sample was measured spectrophotometrically at 520 nm on a microplate reader (SmartReader 96, Accuris Instruments, Edison, NJ, USA). The scavenging potential was expressed as µmol of Trolox per 1 mL of plant extract using a calibration curve.

#### 2.6.3. Ascorbic Acid Content

The content of ascorbic acid (AA) in the fruit extracts was measured using the reflectometric method using an RQ-flex 10 device (Merck, Darmstadt, Germany) according to manufacturer’s instructions for ascorbic acid determination in red berries. The results were expressed as µg/mL of fruit extract.

### 2.7. Antibacterial Potential of Extracts

#### 2.7.1. Agar Well Diffusion Method

The antibacterial potential of blackberry extracts was determined against Gram-positive bacteria, *Staphylococcus aureus* ATCC 25923 and *Bacillus cereus* PCM 482, and Gram-negative ones, *Escherichia coli* NCTC 1893 and *Salmonella enterica* subsp. *enterica serotype* Enteritidis NCTC 12634. For the agar well diffusion method, an aliquot of 0.1 mL of bacterial suspension (prepared from a night culture of bacteria by suspending them until an optical density of 0.132, measured spectrophotometrically at 600 nm) was spread onto MHA (Mueller Hinton Agar, Biomaxima S.A, Lublin, Poland) plates. Then, wells were made using a sterile cork borer (10 mm diameter) and filled with 0.1 mL of appropriately diluted extracts and incubated aerobically at 37 °C for 24 h. Antibiotics (ampicillin and streptomycin, 100 µg/mL) were used as a positive control, and water was used as a negative control [16,17]. The diameters of the inhibition zones were measured in mm after incubation. Each experiment was repeated twice.

#### 2.7.2. Broth Dilution Method

Viable bacterial cell concentrations were estimated by counting CFU’s after 24 h of exposure to the blackberry extracts. *S. aureus* ATCC 25923 and *E. coli* NCTC 1893 were used for this purpose. Fresh night cultures of bacteria grown on MHA (Mueller Hinton Agar Medium, Biomaxima, Poland) were suspended in sterile water to obtain a density of 0.132 when measured spectrophotometrically at 600 nm. The suspension was diluted 150 times (finally the bacterial concentration was around 1.5 × 10^6^ CFU (colony-forming units/mL) [17,18]. Then, serially diluted extracts in TSB (Tryptic Soy Broth medium, Biomaxima, Poland) were prepared. One milliliter of extracts was added to sterile Eppendorf tubes, and 50 µL of bacterial suspension was poured into each tube. Probes were incubated at 37 °C for 24 h without shaking. After incubation serial dilutions were made, and 0.1 mL of serially diluted samples were spread on MHA plates. Plates were incubated for 24 h at 37 °C. After incubation, the colonies formed were counted, and calculated as log_10_ CFU/mL. Moreover, to visualize the antibacterial potential of plant extracts, 20 µL of each dilution was dropped onto the MHA plates in triplicate and incubated for 24 h at 37 °C.

### 2.8. Antiviral Potential of Extracts

Antiviral properties were determined against four bacteriophages with different genetic material: double (ds) and single-stranded (ss) RNA or DNA: phi6 (DSM 21518, dsRNA), host—*Pseudomonas syringae* (DSM 21482); MS2 (DSM 13767, ssRNA), host—*E. coli* (DSM 5695); T7 (DSM 4623, dsDNA), host—*E. coli* (DSM 613); and phiX174 (DSM 4497, ssDNA), host—*E. coli* (DSM 13127). All bacteriophages and their bacterial hosts were purchased from Leibniz-Institute DSMZ, Deutsche SammLung von Mikroorganismen und Zellkulturen GmbH (Braunschweig, Germany). The replication of bacteriophages was performed according to Grabek-Lejko et al. [4]. The antiviral properties of blackberry extracts were determined by using the double agar overlay plaque method. For this purpose, 10 µL of analyzed phage was mixed with 100 µL of appropriately diluted plant extract and incubated at room temperature for 24 h. As positive controls, phages in STM buffer (50 mM Tris-HCl pH 7.5, 0.1 M NaCl, 8.1 mM MgSO_4_, 0.01% (*w*/*v*) gelatin) were used. After incubation, a serial dilution of samples was prepared in STM buffer and added in the volume of 0.010 mL to 100 µL of overnight culture of bacterial host. *P. syringae* was incubated on TSA (Trypticasein Soy Agar, Biomaxima, Poland) medium at 25 °C, and the bacterial concentration used was 0.25 (OD 600 nm), while *E. coli* hosts were incubated on LBA medium at 37 °C (Luria Bertani Agar, Biomaxima, Poland), and the final bacterial concentration used was 0.115 (measured spectrophotometrically at 600 nm). Then, samples were mixed with 5 mL of semi-solid TSB medium (0.7% agar added for phi6 bacteriophage) and with semi-solid LB medium (Luria Bertani, Biomaxima, Poland) (0.7% agar addition for MS2, T7 and phiX174 bacteriophages). The samples were poured onto the TSA plates (for phi 6) and LBA plates (for other bacteriophages) and incubated at 25 °C (for phi 6) and 37 °C (for other bacteriophages). After 24 h of incubation, plaques were counted, and the number of viable bacteriophage particles (able to form plaques) per ml of sample was calculated and expressed as log_10_ PFU (plaque-forming units)/mL. The antiviral potential of plant extracts was analyzed by comparing the analyzed samples with the control samples (pure bacteriophages in SMG buffer, without plant extract).

### 2.9. Statistical Analysis

All measurements were carried out in triplicate. The significance of differences was tested after ANOVA analysis using Tukey’s post hoc test (*p* = 0.05). All calculations were made using Statistica 13.3 software (StatSoft, Tulsa, OK, USA).

## 3. Results and Discussion

### 3.1. pH Value

**Figure 2:**
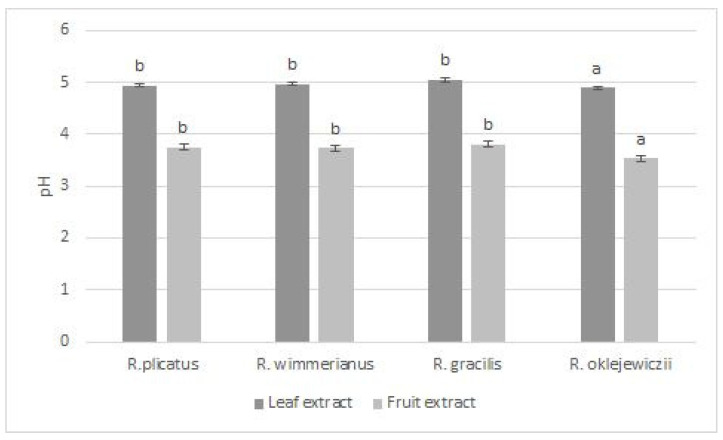
pH values of tested extracts. a,b—means sharing the same superscript are not significantly different (*p* > 0.05) (Tukey’s post hoc test).

The pH values of the analyzed extracts are presented in Figure 2. The obtained results were lower for fruit extracts due to the presence of various organic acids [19]. Similar values for fruits in the range of 2.85—4.066 were reported by Memete et al. [20]. In the case of *R. oklejewiczii* extracts, a significantly lower pH was observed.

### 3.2. Polyphenolic Content and Antioxidant Properties

Leaves were characterized by a higher content of phenolic compounds and antioxidant properties than fruits for all species examined (Table 2). The same tendency were previously observed by Grabek-Lejko et al. [4]. Among the analyzed species the highest phenolic content and antioxidant properties were observed for *R. wimmerianus* fruit and leaf extracts, which was also reported by Oszmianski et al. [7,8]. *R. oklejewiczii* was characterized by the lowest phenolic content and antioxidant potential among all analyzed species. Differences in antioxidant potential and phenolic content of different species of blackberries were also reported by Memete et al. [20]. In the case of leaf extracts, the high content of phenolic compounds translates into higher reducing and antiradical activity. The FRAP and DPPH tests results strongly correlate with the content of phenolic compounds (r = 0.994 and 0.995, respectively). Among the phenolic compounds of leaves, tannins are probably dominant; according to previous data, they constitute over 50% of the total content, and their particularly high content was recorded for *R. wimmerianus* [7]. In the case of fruit extracts, the dominant fraction are anthocyanins, which are responsible for the dark color. The highest contents were determined for the *R. plicatus* extract, and the lowest was for *R. gracilis*.

The determined content of ascorbic acid in blackberry fruit extracts ranged from 192 ug/mL for *R. pllicatus* to 328 µg/mL for *R. oklejewiczii*. In the scientific literature, the results of vitamin C content are given mainly in mg per ml of juice or 100 g of fresh fruit weight and are within the range of 11.9–23.4 (mg/100 g f.w.) [21,22]; hence, it is difficult to compare them with the results obtained for the extract from freeze-dried fruits.

### 3.3. Antibacterial Properties

#### 3.3.1. Agar Well Diffusion Method

The strongest inhibition of bacterial growth of leaf extracts was observed for Gram-positive bacteria, especially for *S. aureus* (Table 3). The weaker antibacterial properties were observed against Gram-negative bacteria, like *E. coli* and *S. enterica*. Comparing the results among blackberry species, the leaf extracts of *R. oklejewiczii* did not inhibit the growth of *B. cereus*, *E. coli*, nor *S. enterica*, while the growth of *S. aureus* was slightly inhibited. Fruit extracts were most effective against *E. coli*, while the weaker bacterial growth inhibition was observed for other analyzed bacteria. *R. oklejewiczii* fruit extract also slightly inhibited the growth of *B. cereus*, while no antibacterial potential was observed for other analyzed bacteria.

The weaker antibacterial properties of *R. oklejewiczii* may be related to the lower content of polyphenolic compounds and, at the same time, the lower values of the FRAP reducing potential, especially in the case of leaf extracts, which significantly differed from the others in terms of polyphenol content. It has been repeatedly demonstrated that the antimicrobial properties of plants correlate with their phytochemical profile, especially polyphenol content [23,24,25]. However, it has also been shown for extracts from cultivated and wild blackberries that antimicrobial activity is not always proportional to the polyphenol content [26]. Other non-phenolic bioactive compounds present in extracts may also be important for this effect, e.g., from the group of terpenoids or organic acids [25].

Antibacterial activity expressed as the diameters of growth inhibition zones correlated significantly with the total content of phenolic compounds in the case of *B. cereus* and *S. enterica* (with correlation coefficients of 0.996 and 0.994, respectively). In the case of these bacteria, a strong correlation with the reducing power of FRAP was also determined, which may indicate the involvement of phenolic compounds and antioxidant mechanisms in inhibiting bacterial growth. However, no significant correlation was observed for the antibacterial properties of and anthocyanin content in fruit extracts. It is known that compounds from this group, characteristic of various berry fruits, have antimicrobial properties [27,28,29], but various factors influencing this effect should be taken into account, such as acidity, type of solvent, as well as synergistic and antagonistic interactions. Water extracts were tested, which may have an important impact on their biological effects. It was previously shown that methanol extracts have the highest content of particular groups of bioactive substances, while water and petroleum ether have the lowest content [30]. It can be speculated that simple organic acids (citric, malic, succinic, fumaric, and oxalic) and also ascorbic acid may be important for biological activity in fruits of *Rubus*, in addition to phenolic compounds [19,31,32,33]. In turn, an important group of metabolites found in the leaves of *Rubus* are tannins, especially the group of gallotannins and ellagitannins, which should be also considered as a powerful antibacterial agents, and its antibacterial potential in blackberries should be analyzed in the future [8,34].

#### 3.3.2. Broth Dilution Method

##### Antibacterial Properties of Leaf Extracts

**Figure 3:**
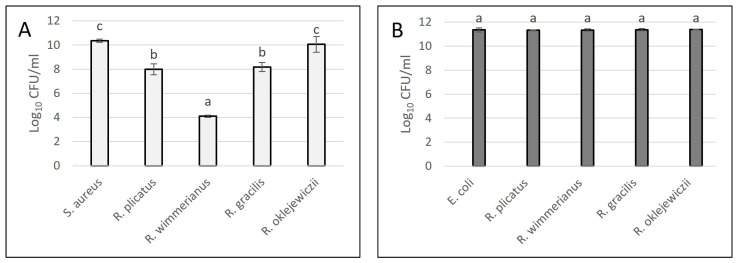
Bacterial growth of *S. aureus* (**A**) and *E. coli* (**B**) after 24 h incubation with leaf extracts (3%). a,b,c—means sharing the same letters are not significantly different (*p* > 0.05) (Tukey’s post hoc test). *S. aureus* and *E. coli* bars—positive control of bacterial growth without addition of plant extract.

The influence of 3% leaf extracts on the growth of *S. aureus* and *E. coli* was shown in Figure 3. After 24 h of contact with viable *S. aureus* cells, an antibacterial effect was observed for all analyzed extracts in comparison with the positive control sample (bacteria without addition of extracts). In the positive control, the bacterial growth reached the level of around 10 log_10_ CFU/mL, while in the case of *R. wimmerianus*, the *S. aureus* growth was strongly reduced by the added leaf extract (bacterial growth at the level of 4 log_10_ CFU/mL). The lowest reduction in the cell viability was observed for *R. oklejewiczii* extract. No inhibition of *E. coli* growth was observed for all tested 3% leaf extracts.

**Figure 4:**
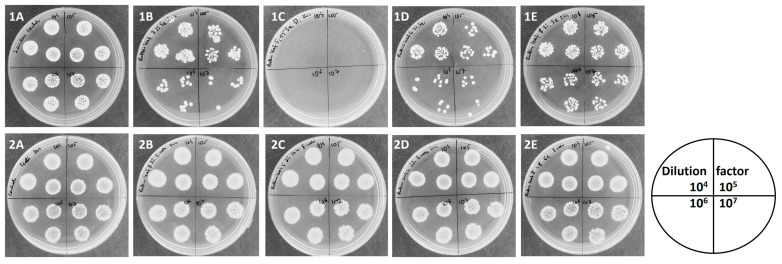
Antibacterial potential of blackberry leaf extracts (3%) against *S. aureus* (**1**) and *E. coli* (**2**) after 24 h of incubation. Upper row from the left: (**1A**)—control sample—*S. aureus* growth; (**1B**)—*R. plicatus* extract; (**1C**)—*R. wimmerianus* extract; (**1D**)—*R. gracilis* extract: (**1E**)—*R. oklejewiczii* extract. Sample dilutions: 10^4^, 10^5^, 10^6^, 10^7^. Lower row from the left: (**2A**)—control sample—*E. coli* growth; (**2B**)—*R. plicatus* extract; (**2C**)—*R. wimmerianus* extract; (**2D**)—*R. gracilis* extract; (**2E**)—*R. oklejewiczii* extract. Sample dilutions: 10^4^, 10^5^, 10^6^, 10^7^.

These results are also visualized in Figure 4. After the bacterial incubation with analyzed extracts, the samples were diluted and, in the form of drops, poured onto the agar medium. After incubation, bacterial growth or a reduction in bacterial growth was observed.

By comparing bacterial growth in Figure 4(1A–1E,2A–2E) it can be observed that the antibacterial potential of blackberry leaf extracts (determined for 3% concentration of extracts) was stronger against *S. aureus* than against *E. coli* (no bacterial inhibition was observed for *E. coli*).

As presented in Figure 4, the strongest anti-stapylococcal activity was determined for *R. wimmerianus* (no bacterial growth) (Figure 4(1C)); weaker activity was observed for *R. plicatus* and *R. gracilis* (the number of bacterial colonies was reduced in comparison with the control sample) (Figure 4(1B,1D)); and the weakest bacterial inhibition was observed for *R. oklejewiczii* extract (Figure 4(1E)). The inhibition of *E. coli* growth was not observed for these extracts.

Antibacterial effect of higher leaf concentrations was also analyzed. Leaf extracts at 25% and 50% concentration totally inhibited *S. aureus* growth, with one exception—in the presence of *R. oklejewiczii* leaf extract, the growth of *S. aureus* was slightly reduced in comparison with the control (at 25% concentration of extract, there was a 2.05 log reduction, and at 50% concentration, bacterial growth was reduced by around 3.07 log). Similar results were observed for *E. coli* growth. The analyzed leaves extracts from tetraploids totally inhibited *E. coli* growth at a concentration of 25% and 50%, while the *R. oklejewiczii* extract reduced the growth of *E. coli* by 1.35 and 2.38 logs, respectively.

##### Antibacterial Potential of Fruit Extracts

**Figure 5:**
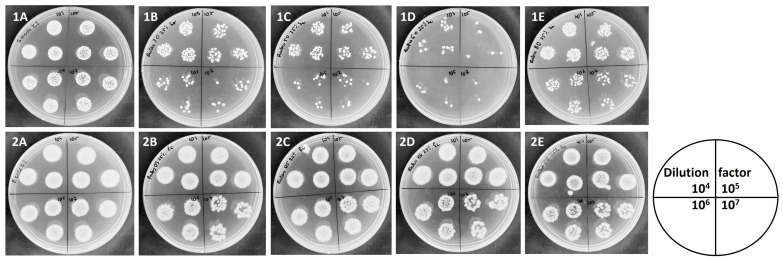
Antibacterial potential of blackberry fruit extracts (25%) against *S. aureus* (**1**) and *E. coli* (**2**) after 24 h of incubation. Upper row from the left: (**1A**)—control sample—S. aureus growth; (**1B**)—*R. plicatus* extract; (**1C**)—*R. wimmerianus* extract, (**1D**)—*R. gracilis*; (**1E**)—*R. oklejewiczii* extract. Sample dilutions: 10^4^, 10^5^, 10^6^, 10^7^. Lower row from the left: (**2A**)—control sample—*E. coli* growth; (**2B**)—*R. plicatus* extract; (**2C**)—*R. wimmerianus* extract; (**2D**)—*R. gracilis*; (**2E**)—*R. oklejewiczii* extract. Sample dilutions: 10^4^, 10^5^, 10^6^, 10^7^.

The antibacterial potential of fruit extracts depended on blackberry species. Generally, the strongest antibacterial properties were observed for *S. aureus* (Figure 5(1A–1E)), than for *E. coli* (Figure 5(2A–2E)). Fruit extracts have weaker antibacterial properties than blackberry leaf extracts. Comparing the antibacterial properties of fruit extracts against *S. aureus*, it was shown that the *R. gracilis* extract had the strongest bactericidal properties (Figure 5(1D)), while the *R. oklejewiczii* extract had the weakest (Figure 5(1E)). *R. plicatus* and *R. wimmerianus* inhibited the growth of *S. aureus* in a comparable manner at the tested concentration of 25%.

Weli et al. [25] demonstrated that the total number of extracts produced from the leaves of blackberry (*R. fruticosus* agg.) expressed moderate to strong antibacterial activity against Gram-positive and Gram-negative bacteria. However, they did not observe the stronger inhibition of Gram-positive bacteria, like *S. aureus.* On the other hand, Jazic et al. [26] assayed the antimicrobial activity of blackberry fruit extracts against *S. aureus* and *E. coli*, and in general, *S. aureus* was more susceptible than *E. coli*. Similarly, Radovanovic et al. [35] studied the antimicrobial activity of wild blackberry extracts against different Gram-positive and Gram-negative bacteria. The MBC (Minimal Bactericidal Concentration) for all tested bacteria ranged from 62.5 μg/mL for *S. enteritidis* and *S. aureus* to 500 μg/mL for *E. coli*, *Klebsiella pneumoniae*, *Proteus vulgaris*, *Clostridium perfringens*, *Bacillus subtilis*, and *Listeria inocua*.

Generally, the strongest antibacterial potential was demonstrated against Gram-positive bacteria compared to Gram-negative ones. This can be related to the presence of a bacteria cell membrane with LPS (lipopolysaccharides) in Gram-negative bacteria, which provides hydrophilic protection, making the penetration of phenolic compounds and terpenoids across the bacteria cell membrane difficult to exert the antimicrobial effect [5].

### 3.4. Antiviral Potential

#### 3.4.1. Antivivral Potential of Leaf Extracts

**Figure 6:**
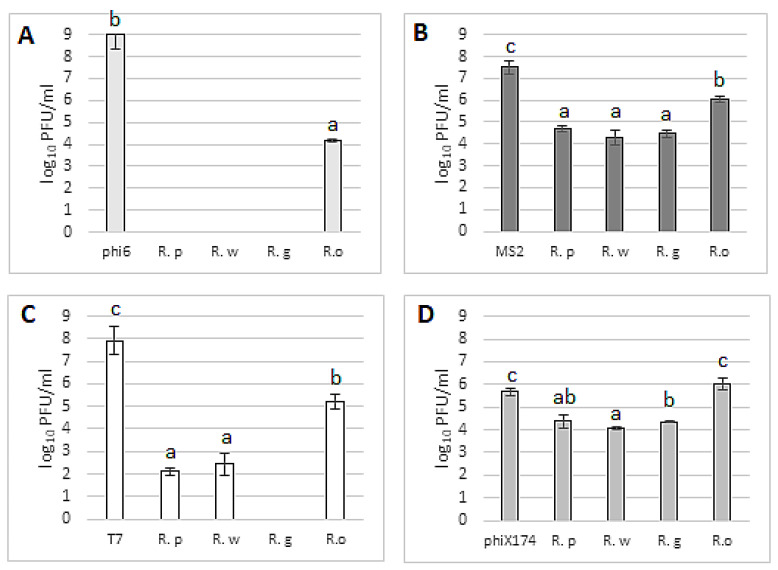
Antiviral potential of blackberry leaves extracts against different bacteriophages: (**A**)—phi6; (**B**)—MS2; (**C**)—T7; and (**D**)—phiX174. Plaque-forming units (PFU) were calculated after 24 h of extract (50%) incubation with bacteriophages. phi6, MS2, T7, phiX174 bars—positive controls (PFU of viral particles in STM buffer without addition of plant extract); R.p—*R. plicatus*; R.w—*R. wimmerianus*; R.g—*R. gracilis*; R.o—*R. oklejewiczii*; a,b,c—means sharing the same letters are not significantly different (*p* > 0.05) (Tukey’s post hoc test); no columns on the graph mean complete virus inhibition by the tested extracts.

The tested blackberry leaf extracts showed varied virucidal effects. Their effectiveness depends on the species of blackberry and the type of virus (Figure 6). The strongest virucidal properties were demonstrated against the bacteriophage phi6 (Figure 6A). At the analyzed concentration, three extracts totally inhibited bacteriophages, while *R. oklejewiczii* leaf extract demonstrated a weaker antiviral potential by reducing the number of virus particles by around 5 log (in comparison with the survival of bacteriophage particles in the sample without the addition of an extract—positive control). The analyzed extracts also very strongly inhibited the bacteriophage T7 (Figure 6C), with total viral inhibition observed for *R. gracilis* extract, while the extracts from *R. wimmerianus* and *R. plicatus* inhibited the virus to a similar extent, reducing the number of viral particles by approximately 6 log compared to the control. The weakest antiviral potential was observed for the *R. oklejewiczii* extract (Figure 6C) (PFU reduction of almost 3 log). The most resistant bacteriophage was phiX174 (Figure 6D). No virucidal potential was observed for the *R. oklejewiczii* extract, while other leaf extracts of blackberries reduced viral particles by around 1.3–1.6 log (in comparison with the control sample). Based on the obtained results, the *R. oklejewiczii* leaf extracts showed the weakest virucidal properties among tested brambles. We can speculate that phenolic compounds play an important role in the antiviral potential of leaf extracts; the lowest amount was observed in the *R. oklejewiczii* bramble. The antiviral potential of phenolic compounds was described previously by Kowalczyk et al. [36].

#### 3.4.2. Antiviral Potential of Fruit Extracts

**Figure 7:**
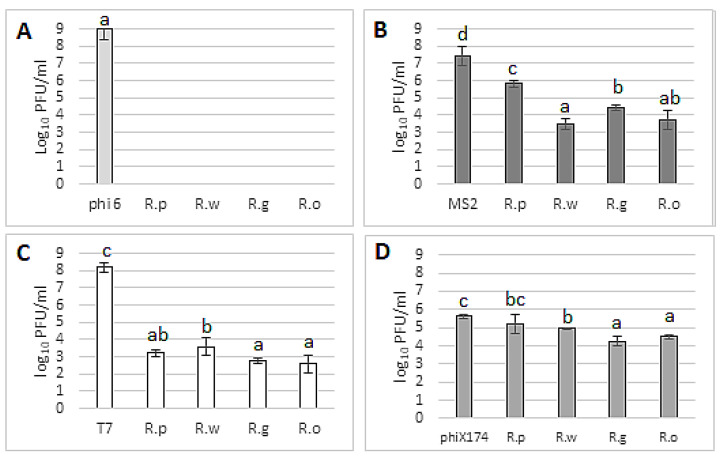
Antiviral potential of blackberry fruit extracts against different bacteriophages: (**A**)—phi6; (**B**)—MS2; (**C**)—T7; and (**D**)—phiX174. Plaque-forming units (PFU) were calculated after 24 h of extract (at 50% extract concentration) incubation with bacteriophage. phi6, MS2, T7, phiX174 bars—positive controls (PFU of viral particles in STM buffer without addition of plant extract); R.p—*R. plicatus*; R.w—*R. wimmerianus*; R.g—*R. gracilis*; R.o—*R. oklejewiczii*; a,b,c,d—means sharing the same letters are not significantly different (*p* > 0.05) (Tukey’s post hoc test); no columns on the graph mean complete virus inhibition by the tested extracts.

The antiviral properties of blackberry fruit extracts are shown in Figure 7. It was demonstrated that the extracts inhibit the tested viruses in various ways. They showed the strongest antiviral properties against the bacteriophage phi6 (as was the case with leaf extracts). At the tested concentration, all extracts completely inhibited the survival of viral particles after 24 h of contact (Figure 7A). Equally strong antiviral properties were demonstrated against the bacteriophage T7 (Figure 7C). In this case, the reduction in viral particles was comparable for all tested extracts, which inhibited the development of viral particles by more than 5 logarithmic units (compared to the control) (Figure 7C). In the case of the bacteriophage MS2 (Figure 7B), different effects of extracts were observed. The strongest viral inhibition was observed for *R. wimmerianus* and *R. oklejewiczii*. The extract of *R. plicatus* fruits had the weakest virucidal properties (a reduction in viable viral particles by approximately 1.6 log). As in the case of the leaf extracts, the most resistant bacteriophage was phiX174 (Figure 7D). The most potent extracts were from the fruits of *R. oklejewiczii* and *R. gracilis*, which reduced the number of viral particles by approximately 1.1–1.4 logs, while the extracts from *R. plicatus* did not reduce the number of viable viral particles. The resistance of used bacteriophages for leaf and fruit extracts was similar, and the highest resistant was observed for phiX174, while the lowest was observed for phi6 bacteriophages.

**Figure 8:**
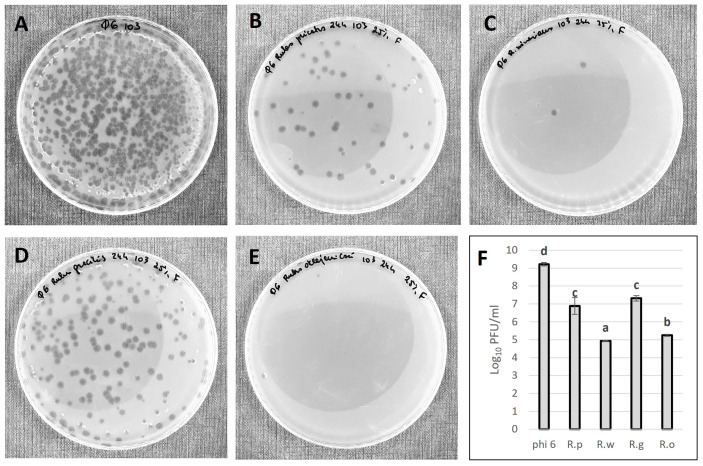
Antiviral potential of blackberry fruit extracts against phi6 bacteriophage. Plagues generated after 24 h of 25% fruit extract incubation with phi6. (**A**)—control plate, phi6 in STM buffer; (**B**)—*R. plicatus*; (**C**)—*R. wimmerianus*; (**D**)—*R. gracilis*; (**E**)—*R. oklejewiczii*. Dilution factor: 10^3^. (**F**)—Plaque-forming units (PFU) were calculated after 24 h of extract (at 25% concentration) incubation with bacteriophage. R.p—*R. plicatus*; R.w—*R. wimmerianus*; R.g—*R. gracilis*; R.o—*R. oklejewiczii*; a,b,c,d—means sharing the same letters are not significantly different (*p* > 0.05) (Tukey’s post hoc test).

In order to distinguish the antiviral potential of fruit extracts against the bacteriophage phi6, lower concentrations of extracts (25%) were analyzed. In this case, the highest inhibition of phi6 particles was observed for the *R. oklejewiczii* and *R. wimmerianus* extracts (Figure 8). We cannot compare our results with other authors because of the lack of such data. In our previous work [4], we demonstrated that blackberry fruits and leaves of mixed species (water–methanolic extracts) possessed antiviral potential against the bacteriophage phi6, reducing the viral particles by around 4 log_10_ PFU/mL.

Generally, the fruit extract of the pentaploid *R. oklejewiczii* had the highest antiviral potential. This fruit extract is characterized by a higher content of ascorbic acid, significantly lower pH, and the lowest phenolic content and antioxidant properties. This may suggest that the antiviral potential of blackberry fruits can be associated with pH and ascorbic acid content. A negative correlation (−0.692) between the content of vitamin C and the number of viable viral particles of bacteriophage phi6 was determined. The antiviral potential of ascorbic acid and low pH was described previously [37,38]. Ascorbic acid is a crucial vitamin for the correct functioning of the immune system of organisms. There is evidence that vitamin C and quercetin (one of the polyphenols which is also present in blackberry fruits [8,20]) also exhibit a synergistic antiviral effect against the SARS-CoV-2 virus. These compounds may be present in blackberry fruit extracts. Quercetin and vitamin C may disrupt virus entry and replication, simultaneously fortifying the immune response of the organism [39,40,41].

The strong antiviral potential of the analyzed bramble extracts against the bacteriophage phi6 is very important. The bacteriophage phi6 is a virus that belongs to a different Baltimore group than SARS-CoV-2 (group III instead of IV). However, it has a round-like shape and a lipid envelope like SARS-CoV-2, which render it very useful as a surrogate of this infectious pathogen for biosafety reasons [42]. The obtained results suggest that water extracts of leaves (which can be used in the form of tea in our diet) and fruits can be a good source of antiviral substances and can play a very important role in fighting against SARS-CoV-2. The varied antiviral properties of the tested extracts indicate the need to analyze the antiviral potential against different viruses because the same extracts may or may not have reduced the viability of viral particles. It was not possible to observe a clear relationship between the content of polyphenols and antioxidant activity, and the antiviral effect of the tested extracts in all cases, but some tendency can be observed. The lowest antiviral potential of leaf extracts was observed in the bramble with the lowest phenolic content. In fruits, it is probable that other compounds (like ascorbic acid or other organic acids, low pH) are involved in this action, which would suggest a different type of mechanism and the need for further analysis.

In order to fully explain the antibacterial and antiviral effect of the tested extracts, a detailed phytochemical analysis is necessary, which will allow for the linking of the content of metabolite groups, and even individual compounds, with the biological effect.

### 3.5. Ploidy Level and Biological Properties

Polyploidy is a crucial feature in vegetative and generative growth as it affects plant development. Thus, the effects of polyploidy deserve to be evaluated on fruit set and seed production in breeding. The duplication of the genome, and its possible adaptive advantages, has been an important factor in the speciation and evolution of eukaryotes. Compared to diploid plants, tetraploids are sometimes characterized by larger fruits, a lack of seeds, higher plant production, and changes in the metabolism of secondary metabolites [43,44]. Ploidy levels among different subgenera and species of *Rubus* are highly differentiated, and the duplication of the genome is very important for the ability to occupy various habitats worldwide [45].

Despite the importance of ploidy level in plants, there is only limited information [46] concerning the variation in bioactive compounds within and between *Rubus* species with different ploidy levels, and their association with fruit quality traits, which makes it difficult to compare the results obtained in this study with other authors’ findings.

Sabooni et al. [47] demonstrated that ploidy level correlated significantly with the total soluble solids in the leaves and with the number of pistils, leaf green index, and glucose content in floral nectar in blackberries (*R. persicus*, *R. caesius*, and *R. sanctus*). However, the content of polyphenols was not correlated with ploidy level but did depend on the bramble species and ploidy level. For example, in *R. persicus*, the polyphenol content in diploids, tetraploids, and octaploids were 175, 155, and 222 mg of GAE/L, respectively. In *R. caesius* and *R. sanctus*, higher ploidy lead to lower antioxidant potential and polyphenolic content. In the case of *R. oklejewiczii*, higher ploidy, compared to *R. gracilis*, *R. plicatus*, and *R. wimmerianus*, did not correlate with a higher polyphenolic content nor antioxidant potential, even in the case of the leaves in relation to antibacterial and antiviral potential. Li and Hoshino [48] and Fujita et al. and [49] analyzed the impact of ploidy level (2x, 4x, and 6x) in haskap (*Lonicera caerulea*) fruits on its biochemicals content. They observed a strong positive linear correlation between ploidy level and specific substances, like quinic acid and sorbitol, and the accumulation of some sugars (glucose and fructose). On the other hand, several other substances had little or weak linear correlation with ploidy. Moreover, tetraploid fruits demonstrated superior quality compared to other ploidy levels. A higher content of bioactive substances in tetraploid brambles in comparison with pentaploid *R. oklejewiczii* species was also observed in our work.

Mengist et al. [50] compared the bioactive compound concentrations and fruit quality in blueberry fruits with different ploidy level (which, similar to *Rubus* fruits, belong to the so-called “superfruits” group). They observed that tetraploid plants had a higher pH, acylated anthocyanins, and flavonol concentrations than diploid and hexaploid ones. On the other hand, the highest content of non-acylated anthocyanins and flavanols was observed for diploid plants. The total phenolic acid content was lowest for tetraploids, while the level of phenolic acids was comparable between the diploid and hexaploid groups. In *Salvia officinalis* tetraploids, phenol and flavonoid content was higher than in diploids, while antioxidant properties were comparable [51].

These results emphasize the complex and sometimes unexpected outcomes of polyploidization, which was also concluded by Allario et al. [52]. It means that the “polyploid effect” can influence the secondary metabolite production pathway differentially. The ploidy level produces changes in the regulation of the synthesis of metabolites. Consequently, while certain metabolites are enhanced, others are diminished in favor of the former [53].

Therefore, emphasis is placed on the importance of expanding research with detailed analyses of individual substances in various species of *Rubus*, differing in ploidy, in order to fully understand and broaden the knowledge of this under-explored group of angiosperms. This is required to fully exploit the potential of polyploids plants in breeding and support nutritional and economic benefits.

## 4. Conclusions

*R. oklejewiczii*, a recently described new bramble, exhibits antibacterial, antiviral, and antioxidant properties. However, the increased polyploidy of the plant does not affect the better biological properties of leaf extracts compared to the tested extracts of 4-ploid species. Similarly, *R. oklejewiczii* fruit extracts showed the weakest antioxidant properties and the lowest polyphenol content; however, the antiviral properties were stronger, which correlated with the lowest pH and the highest ascorbic acid content, suggesting the possibility of using fruits of this species as a potential rich source of antiviral substances and ascorbic acid in our diet.

Despite the fact that most biological properties of the tested *R. oklejewiczii* extracts did not stand out compared to other examined species, due to its large and tasty fruits, *R. oklejewiczii* seems to be a very promising species for cultivation and attractive to consumers and producers.

Additional detailed analyses of the phytochemical composition and its effect on biological properties are necessary in the future, also taking into account other species of blackberries.

## Figures and Tables

**Table 1:** List of populations examined.

Species	Voucher Specimens Number	Ploidy Level	Geographical Location		Time of Harvesting
*R. plicatus*	003724a,b	4x	Piątkowa	N 49°52′53.6″; E 022°08′ 11.3″	19.07.2022
*R. wimmerianus*	003710a,b	4x	Lecka–Wilczak	N 49°53′12.0″; E 22°01′ 09.2″	26.07.2022
*R. gracilis*	003711a,b	4x	Lecka–Wilczak	N 49°53′12.9″; E 22°01′ 10.0″	26.07.2022
*R. oklejewiczii*	003705a,b	5x	Kąkolówka–Wola	N 49°51′16.5″; E 22°01′ 39.8″	28.07.2022

**Table 2:** Phenolic content and antioxidant potential of blackberry fruit and leaves extracts.

Blackberry Extract	Leaf Extracts			Fruit Extracts				
	TPC (µg/mL)	DPPH (µmol/mL)	FRAP (µmol/mL)	TPC (µg/mL)	TAC (µg/mL)	DPPH (µmol/mL)	FRAP (µmol/mL)	AA (µg/mL)
*R. plicatus*	1026.67 ± 42.59 ^c^	41.34 ± 0.66 ^b^	12.92 ± 1.13 ^c^	119.44 ± 6.38 ^c^	113.86 ± 0.47 ^d^	9.71 ± 1.00 ^a^	1.69 ± 0.04 ^d^	268 ± 11 ^a^
*R. wimmerianus*	1097.22 ± 42.80 ^d^	42.08 ± 0.06 ^b^	13.56 ± 1.34 ^c^	105.00 ± 2.13 ^bc^	105.85 ± 6.14 ^c^	12.24 ± 0.66 ^b^	1.53 ± 0.09 ^c^	302 ± 8 ^b^
*R. gracilis*	805.00 ± 19.40 ^b^	41.94 ± 0.15 ^b^	10.31 ± 0.80 ^b^	98.89 ± 2.22 ^b^	69.28 ± 2.60 ^a^	10.94 ± 0.80 ^a^	1.41 ± 0.05 ^b^	292 ± 20 ^ab^
*R. oklejewiczii*	271.11 ± 5.13 ^a^	24.05 ± 0.75 ^a^	1.87 ± 0.13 ^a^	80.00 ± 15.71 ^a^	76.13 ± 0.94 ^b^	11.71 ± 0.21 ^b^	1.01 ± 0.02 ^a^	328 ± 27 ^b^

^a,b,c,d^—means sharing the same superscript are not significantly different (*p* > 0.05) (Tukey’s post hoc test). TPC—total phenolic content (expressed as gallic acid equivalents); TAC—total anthocyanin content (expressed as cyanidin-3-glucoside equivalents); DPPH, FRAP—expressed as Trolox equivalents; AA—ascorbic acid.

**Table 3:** Antibacterial properties of blackberry extracts using agar well diffusion method. Results in mm of inhibition.

Rubus	Leaf Extracts				Fruit Extracts			
	Gram-Positive		Gram-Negative		Gram-Positive		Gram-Negative	
	S.a	B.c	E.c	S.e	S.a	B.c	E.c	S.e
*R. plicatus*	21 ± 0.14 ^b^	17 ± 0.06 ^c^	15 ± 0.07 ^d^	14 ± 0.07 ^c^	13 ± 0.14 ^b^	12 ± 0.10 ^b^	15 ± 0.18 ^b^	11 ± 0.07 ^b^
*R. wimmerianus*	22 ± 0.00 ^c^	17 ± 0.07 ^c^	12 ± 0.12 ^b^	15 ± 0.04 ^d^	14 ± 0.10 ^c^	11 ± 0.12 ^a^	15 ± 0.00 ^b^	11 ± 0.00 ^b^
*R. gracilis*	23 ± 0.07 ^d^	15 ± 0.21 ^b^	13 ± 0.07 ^c^	13 ± 0.08 ^b^	14 ± 0.12 ^c^	15 ± 0.14 ^d^	18 ± 0.20 ^c^	15 ± 0.06 ^c^
*R. oklejewiczii*	12 ± 0.07 ^a^	10 ± 0.00 ^a^	10 ± 0.00 ^a^	10 ± 0.00 ^a^	10 ± 0.00 ^a^	13 ± 0.50 ^c^	10 ± 0.00 ^a^	10 ± 0.00 ^a^
Ampicillin	45 ± 0.00	19 ± 1.2	40 ± 0.22	40 ± 0.24	-	-	-	-
Streptomycin	35 ± 0.00	35 ± 2.3	35 ± 0.25	40 ± 0.32	-	-	-	-
Water	10 ± 0.00	10 ± 0.00	10 ± 0.00	10 ± 0.00	10 ± 0.00	10 ± 0.00	10 ± 0.00	10 ± 0.00

S.a—*Staphylococcus aureus*; B.c—*Bacillus cereus*; E.c—*Escherichia coli*; S.e—*Salmonella enterica*; ^a,b,c,d^—means sharing the same superscript are not significantly different (*p* > 0.05) (Tukey’s post hoc test).

## Data Availability

Data is contained within the article.
